# Graduate student confidence following a for-credit systematic review course pilot

**DOI:** 10.5195/jmla.2021.1073

**Published:** 2021-04-01

**Authors:** Bethany Sheriese McGowan, Jason B. Reed, Jane Kinkus Yatcilla

**Affiliations:** 1 bmcgowa@purdue.edu, Associate Professor and Health Sciences Information Specialist, Purdue University, West Lafayette, IN; 2 reed252@purdue.edu, Purdue University, West Lafayette, IN; 3 janeyat@purdue.edu, Purdue University, West Lafayette, IN

**Keywords:** systematic review, instruction, student-centered learning, faculty librarians

## Abstract

**Background:**

In 2015, librarians at Purdue University began fielding requests from many disciplines to consult or collaborate on systematic review projects, and in 2016, health sciences librarians led the launch of a formal systematic review service. In 2019, Purdue University Libraries was reorganized as the Libraries and School of Information Studies (PULSIS) and assigned its own course designation, ILS. The increase in calls for systematic review services and the ability to teach ILS courses inspired the development of a credit-bearing ILS systematic review course.

**Case presentation:**

We designed, taught, and assessed a one-credit systematic review course for graduate students, using a backward-design course development model and applying self-determination theoretical concepts into lessons, assignments, and assessments. Using qualitative pre- and post-assessments, we discovered a variety of themes around student motivations, expectations, and preferences for the course. In quantitative post-class assessments, students reported improved confidence in all systematic review processes, with the highest confidence in their ability to choose and use citation management managers, describe the steps in the systematic review process, and understand the importance of a reproducible and systematic search strategy.

**Conclusions:**

We considered our pilot a success. Next steps include testing 2- and 3-credit- hour models and working to formally integrate the course into departmental and certificate curriculums. This case report provides a model for course design principles, learning outcomes, and assessments that librarians and library administrators can use to adjust their systematic review services.

## BACKGROUND

Purdue University is a land grant institution in Indiana, with a student body of more than 40,000, of which 75% are undergraduates, 23% are graduate students, and 2% are professional students. While Purdue does not have a medical or dental school, there is a considerable amount of clinical and health-related teaching and research conducted on campus through the Colleges of Pharmacy, Veterinary Medicine, and Health and Human Sciences (HHS). HHS includes the School of Health Sciences and the School of Nursing as well as the Departments of Health and Kinesiology, Human Development and Family Studies, Nutrition Science, Psychological Sciences, Public Health, and Speech, Language, and Hearing Sciences.

Around 2015, Purdue Libraries faculty began receiving requests for systematic review assistance from graduate students within and outside the health sciences. Many students were either (1) taking a course in which they were expected to conduct a systematic review or (2) expecting to play a prominent role in a systematic review project led by a faculty member.

In response to the increase in systematic review consultation requests, librarians attended systematic review training workshops at the University of Pittsburgh and the University of Michigan. A formal systematic review service was launched in 2016, led by a team of health sciences librarians. In 2019, Purdue University Libraries was reorganized as the Purdue University Libraries and School of Information Studies (PULSIS) and assigned its own course designation, ILS. This allowed Libraries faculty, who had previously been required to co-teach courses with other disciplinary faculty or under other departments’ designations, to design and teach PULSIS-owned courses. These two shifts, an increase in calls for systematic review services and the ability to teach ILS courses, led to the development of a credit-bearing ILS systematic review course.

The demand for systematic review services in academic libraries is significant, and librarians work on systematic reviews in a range of roles [1, 2]. Cochrane, the Institute of Medicine, and the Medical Library Association recommend that systematic review teams include a librarian or information specialist [3–6].

While there are no studies specifically describing a librarian-designed and -taught for-credit course on systematic reviews, the literature shows evidence of how librarians have applied their professional expertise to help students and practitioners develop the comprehensive searching skills required for evidence synthesis projects. Systematic review classes taught by librarians have typically focused on searching [7, 8] and citation management skills [9], while for-credit courses taught by non-librarian instructors have used a variety of intensive teaching methods to produce complete reviews at the completion of the course [10, 11].

We report on the development and launch of a new graduate level, credit-bearing course, Introduction to Systematic Review for the Health Sciences, co-designed by three PULSIS faculty librarians.

## CASE PRESENTATION

### Course structure

Given that we were teaching an elective course to an audience of busy graduate students, we opted for a one-credit-hour course pilot over eight weeks, with twice-weekly meetings (50 minutes each class).

### Course development

Course development was based on a backward design course development model [12]. In the backward design model, instructors begin with what they want the students to gain from the course, then create assessments that measure whether students succeeded in meeting the course outcomes. We began by establishing the overarching course learning outcomes, then constructed the learning objectives, assignments, and assessments. To develop the course learning outcomes, we leveraged our experiences working on systematic review teams, reviewed the related literature, and interviewed disciplinary faculty experienced with the systematic review process. The learning outcomes for the course were that students would be able, after course completion, to (1) describe the steps in the systematic review process, (2) understand the importance of a reproducible and systematic search strategy, (3) identify bias in health sciences literature, and (4) implement data management strategies.

Next, we determined the best approach for assessing student learning. We used Deci and Ryan's self-determination theory to guide the creation of course assignments and assessments and to inform the delivery of course content [13]. Self-determination theory consists of three pillars of motivation: (1) autonomy—students feel they have choices in the classroom and on assignments; (2) relatedness—students feel connected with class participants and assignments; and (3) competence—students believe they are capable of mastering the content. We implemented a series of scaffolded assignments and assessments that culminated in a final project, the mock registration of a systematic review protocol. Our approach of modeling course assignments on the sections of a systematic review protocol supported autonomy and relatedness by allowing students to self-select research topics and to select alternative review types more suited to their research needs if necessary. Our approach supported student competence by providing students with detailed feedback and guidance through each major phase of the protocol development process.

### Course layout

The course opened in Week 1 with a pre-assessment, an overview of what a systematic review is, and why protocols are important. These points of clarification were especially important for students who thought they would be completing a systematic review during the eight-week course. In Weeks 1–3, the course focused on steps related to developing a search strategy, including selecting a research question framework, developing a research question, selecting relevant databases, building a search string, and developing inclusion and exclusion criteria.

The next series of classes, in Week 4, focused on tools for automating systematic review processes, primarily from a data management perspective. These included a class on citation management software and another on citation screening tools.

The final sessions of instructor-led classes, in Weeks 5 and 6, included lectures on searching for grey literature, assessing the risk of bias, and an introduction to meta-analysis. The sessions on assessing the risk of bias and introduction to meta-analysis were led by guest instructors with expertise in these areas.

At the end of Week 6, we offered a question-and-answer class where students asked outstanding questions about their protocols or about systematic review methodology in general. Students submitted a draft of their protocols and received instructor feedback before the class. Week 7 of the course included two class sessions of student presentations, with each student having 5–7 minutes to present their protocol to the class, answer questions, and receive peer feedback.

On the final day of the course in Week 8, we conducted a debrief session and distributed a post-assessment.

### Course assignments and assessments

Homework assignments were distributed throughout the course, and most were components of the final mock protocol, based on the PROSPERO systematic review protocol structure. These assignments included having students craft their research question, compile a list of relevant databases, construct search strategies, practice using citation management and screening tools, write a draft mock protocol, and submit a final mock protocol. These assignments highlighted the iterative nature of the protocol development process and encouraged students to think through the full review process. Students submitted a mock registration protocol as the final course deliverable and were encouraged to formally submit their protocols if they desired. A full course syllabus is available in the supplemental material.

### Faculty commitments

Three PULSIS faculty librarians, with a combined total of 20 semesters of teaching experience in Purdue's IMPACT program, designed the course over 13 weeks, spending approximately 2 to 3 hours of labor each week. Two faculty [BSM and JBR] co-taught the course pilot, with both attending each class to lend their expertise and gain instruction experience. We alternated grading duties, allowing for consistency within grades while sharing the grading workload.

### Course logistics

We utilized Blackboard, the university's course management system, to manage communication with the class and to manage assignments and grading. We assigned a series of journal articles as course readings that introduced class topics, with one or two articles assigned per class session.

The classes took place in a Libraries-maintained computer lab. The course design plan was based on student-centered learning pedagogies: instructors would spend the first half of each 50-minute class lecturing, then students would spend the latter half on hands-on activities. This did not work as planned because the students were highly engaged and asked questions during the lecture. While this engagement helped everyone think about the topics more critically, the questions usually took up much of the time previously allocated to hands-on activities.

### Class demographics

We actively promoted the course to health sciences graduate programs during the Fall semester, before its Spring launch, using emails, listserv announcements, and physical flyers. We directly recruited students who attended Libraries-led workshops and promoted the course during invited lectures in graduate health sciences classes and labs. Although it was labor intensive, the recruitment effort was a success: nine graduate students enrolled for credit, and one student audited the course. A majority of students were from the HHS/Department of Nutrition Science (n=6), and the remainder were from the Purdue University Polytechnic Institute (College of Technology) (n=2), the College of Agriculture (n=1), and the HHS/School of Health Sciences (n=1).

## SURVEY TOOLS AND DISTRIBUTION

This study was approved by the Purdue University Institutional Review Board (Study #1901021574).

### Survey tools

We used three assessment tools to measure student motivation for course enrollment, student expectations of the course, and student confidence executing systematic review processes at course completion. When assessing student confidence, we asked students to rate both their confidence in their ability to perform a set of skills related to systematic review processes and the extent to which their confidence in performing these skills had improved during the course. The first survey was given on the first day of the course, and the other surveys were given on the last day of the course. Survey instruments are provided in Appendix 1.

### Survey distribution and analysis

All surveys were distributed in print form, and students were provided time in class to complete and return the surveys. Completed surveys were returned to the instructors at the end of the class. The surveys were transcribed, transferred, and stored using Qualtrics survey software.

Responses from the qualitative pre- and post-assessments were manually coded and thematically sorted by the lead author (BSM), using Excel. Because of overlap between responses, we combined responses from the three qualitative pre-assessment questions into a single thematic analysis related to motivation for course enrollment, course expectations, and additional desired content. A comment was defined as any new idea or topic addressed in a response, and a single response could include multiple comments.

We used Excel to analyze data from the quantitative post-assessment survey. Each survey response was assigned a value, as follows: Strongly Agree=5; Agree=4; Neutral=3; Disagree=2; and Strongly Disagree=1. We created a mean response score for each question by calculating the average of response scores each question received.

## SURVEY RESULTS

Ten students enrolled in the course, and nine participants responded to each survey, for a 90% response rate. Participants mostly answered all survey questions. Exceptions included the pre-assessment survey question related to missing syllabus content, which seven students did not answer, and the qualitative post-assessment regarding ideal credit hours, which one student did not answer.

### Qualitative pre-assessment survey results

We identified 13 unique themes in the 38 comments around student motivations and course expectations from the pre-assessment survey results. Popular themes included plans to write a systematic review (6 comments), a desire to learn more about data management (5 comments), a desire to learn to design a search strategy (5 comments), and a desire to understand the steps for conducting a systematic review (5 comments). Additional themes are illustrated in the supplemental material.

### Qualitative post-assessment results

All respondents (n=9, 12 total comments) said the course met their expectations. A thematic coding of responses related to what participants enjoyed most about the course revealed five themes: course structure (7 comments), knowledgeable and open course instructors (2 comments), useful feedback on assignments (1 comment), useful course resources (1 comment), and learned a new skill (1 comment). Three themes emerged when analyzing the nine comments related to what students disliked about the course: nothing (4 comments), wished the course was longer (4 comments), and a preference for lectures from the course instructors over guest lecturers (1 comment) Additional responses related to what participants enjoyed most, disliked, and found more informative about the course are included in the supplemental material.

All respondents (n=9) would recommend the course to others in their program. An analysis revealed two reasons: applicability of course to graduate research (8 comments) and small class size (1 comment).

We asked students if they would take the course if it was longer, especially if it moved from 1 credit hour to 2 credit hours. Of the students who responded, most indicated they would (n=6) or probably would (n=1) take the longer course, and one student would not. We asked students for their thoughts on the ideal number of credit hours for the course. One student thought the course was ideal as one credit hour, while four thought the course would be ideal as two credits. Other responses were mixed, with a variety of disparate suggestions.

Most students (n=6) rated 11:00 a.m. to 1:00 p.m. as the best time for this course. Afternoon, between 1:00 and 3;00, was also a popular preference, receiving 4 responses. Less desirable times were morning (9:00–11:00 a.m.) and late afternoon (3:00–5:00 p.m., which received 2 responses each; no one selected early mornings (8:00–9:00 a.m. or evenings (after 5:00 p.m.). One student reported their preference would depend on their class and lab schedules.

### Quantitative post-assessment survey results

**Figure 1:**
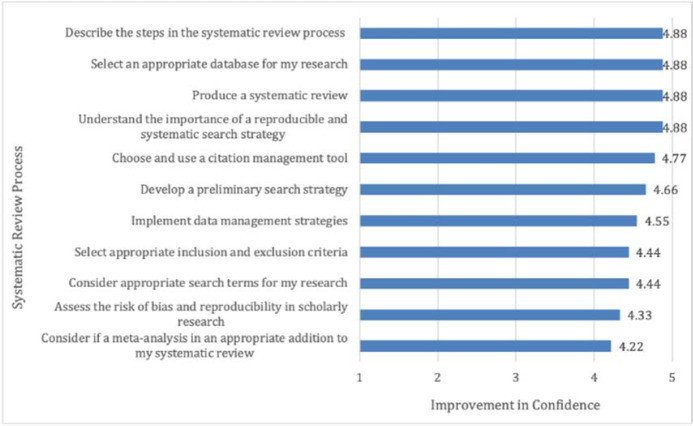
Post-assessment survey results: Student self-reported improved confidence at course end

Results from the post-assessment survey on student confidence showed that students’ self-reported confidence in their abilities to execute systematic review processes had improved by the course's completion (Figure 1). Skills with the highest mean scores were improvement of confidence in the ability to describe the steps in the systematic review process, select appropriate databases, produce a systematic review, and understand the importance of a reproducible and systematic search strategy, all of which showed 4.88 on a 5-point Likert measure. The lowest mean score (4.22) was ability to consider if a meta-analysis was an appropriate addition.

**Figure 2:**
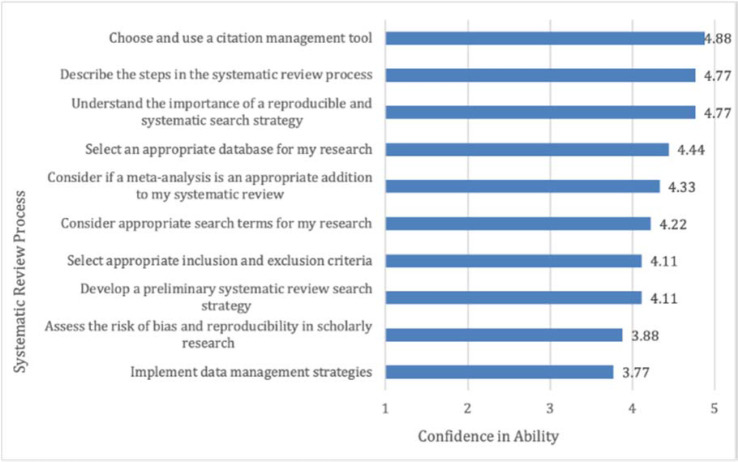
Post-assessment survey results: Student self-reported confidence in systematic review processes at course end

We also asked students to report how confident they felt executing systematic review processes at the end of the course, regardless of any improvements (Figure 2). Students reported the most confidence in their ability to choose and use citation management tools (with a mean response of 4.88) and in their abilities to describe the steps in the systematic review process and to understand the importance of a reproducible and systematic search strategy (each with a mean response of 4.77). Students were much less confident in their abilities to implement data management strategies and assess the risk of bias and reproducibility in scholarly research, with mean responses of 3.77 and 3.88, respectively.

## DISCUSSION

These results suggest that our eight-week one-credit course bolstered student confidence in executing systematic review processes. This finding is consistent with that in the public health course referred to above, where a majority of past students (68.4%) found the course useful in conducting subsequent systematic reviews, and most (93.3%) found the course useful in critically appraising reports of systematic reviews [11]. As of Spring 2020, one year after course completion, seven of the students have led systematic review teams whose findings have been submitted to or been published in journals or been presented at local or national conferences. These results are similar to those found in the course for psychiatric residents, where many of the residents proceeded to present at national meetings and publish peer-reviewed papers based on their course project [10].

Our results measuring the self-reported confidence of student abilities indicate that the pilot was most successful at meeting the first two course learning outcomes: describe the steps in the systematic review process and understand the importance of a reproducible and systematic search strategy. For both competencies, students rated their end-of-course confidence a mean score of 4.77 (out of 5). The course was less successful at meeting the third and fourth outcomes: identify bias in health sciences literature and implement data management strategies, which received mean scores of 3.88 and 3.77, respectively. The most likely reason for this difference is that the third and fourth learning outcomes are higher-level cognitive concepts. While the course was designed to reinforce these more difficult concepts through in-class activities, the instructors rarely had time to complete these activities in practice due to high student engagement during the lectures. Although in-class activities are important, we believe our decision to skip these activities in favor of prioritizing student-led discussions allowed for richer engagement, and we consider our approach a success in that respect. To address the need to balance time for activities and discussion, we recommend increasing the number of credit hours to create more time for in-class activities, which would parallel Li et al.'s [11] course both in terms of use of class time and increased number of credits. Adding an additional credit hour, which students recommended in the debrief session, would allow us to maintain student engagement while also creating time for the planned activities.

The incorporation of Deci and Ryan's [13] self-determination theory helped structure the course and create an engaging experience for the students. While responding that the course met their expectations, several students commented specifically on their appreciation for the course structure, and all said they would recommend the course to a colleague. We promoted autonomy by teaching students to recognize whether a systematic review fit their research needs and offering alternatives if it didn’t, and by allowing students to explore a self-selected research topic. In terms of relatedness, we created a learning environment that encouraged students to participate and ask questions, and we offered detailed feedback on assignments. This resulted in engagement that remained high throughout the course, with robust, student-led discussions. Finally, feelings of competence are illustrated by the success of several course alumni who, one year after course completion, are in the final stages of completing reviews or have already published or presented review findings (several of which involve the course instructors as co-authors and mentors).

In addition to the demonstrated benefits for students, this course strengthened the relationship between PULSIS faculty and campus partners and illustrates how Libraries instruction supports the university's mission of providing transformative learning experiences.

## CHALLENGES, LIMITATIONS, AND NEXT STEPS

Marketing and recruitment for the course have posed the biggest challenges. Although PULSIS faculty teach independently, we rely on students from other departments for course enrollment. We hope to address this challenge by working with faculty and administrators to integrate the course into departmental course curriculums, so students receive formal credits in their departments rather than elective credits. As most graduate plans of study require one or more research courses, this approach could increase course visibility and create an incentive for registration. We do not envision this course becoming a required course in a department's degree program; rather, it would be an approved course that would count toward the number of research course credits required. We might also integrate the course into the curriculum for graduate certificates.

Another challenge is the time and resource investment of the faculty librarians teaching the course. Each librarian committed 2–3 hours per week during the 13-week IMPACT course design model, and two instructors spent an additional 2–3 hours preparing, teaching, and grading for each of the 16 sessions, for a total of 58–87 hours spent by each librarian throughout the course. If this course grows in popularity, we might consider recruiting additional PULSIS faculty as course instructors.

Finally, limitations of our study design prevent us from drawing broad conclusions. Our results relied on students’ ability to accurately report improvements in their self-confidence, using questions that were framed positively and that relied on a Likert measure. Responses may have been prone to confirmation bias. The lack of an equivalent pre-assessment means that the reporting of improvement is subject to reporting bias, as students may have over-estimated improvement in their confidence at the end or conversely, under-estimated their confidence at the beginning. To address this issue, future iterations of the course will include a quantitative pre-assessment, and we will re-consider the framing of the questions in the confidence survey.

## CONCLUSIONS

This systematic review course pilot illustrates how Purdue Libraries and School of Information Studies faculty support the university's mission of providing transformative learning experiences. The use of a backward design course development model, paired with Deci and Ryan's self-determination theory, allowed for the creation of an engaging instructional experience for graduate students. Our results suggest that students enrolled in the course because they planned to conduct a systematic review and wanted to learn the steps in the process. By the end of the course, students reported confidence in describing the steps in the systematic review process as one of their most improved abilities. These results, along with other reported measures, suggest that the course successfully met its learning objectives and student expectations.

## Data Availability

The Student Confidence Survey, the coded datasets for the pre- and post-qualitative assessments, the course syllabus, data tables, and the results of the quantitative post-assessment analysis are available in the Purdue University institutional repository, e-Pubs, at https://docs.lib.purdue.edu/lib_fssup/8.

## SUPPLEMENTAL FILES

**Appendix A:** ILS 595—Introduction to Systematic Review for the Health Sciences

**Appendix B:** Survey Instruments
