# Supplementary material for: Graduate student confidence following a for-credit systematic review course pilot
Source: J Med Libr Assoc. 2021 Apr 1;109(2):323–9. doi: 10.5195/jmla.2021.1073 (PMC8270359; doi:10.5195/jmla.2021.1073)
Supplement: Supplementary file 1 — Appendix A: ILS 595—Introduction to Systematic Review for the Health Sciences [file jmla-109-2-323-s01.docx]

| **APPENDIX 1. SURVEY INSTRUMENTS**  **Tool 1:** A Qualitative Pre-Assessment Survey   1. Why did you sign up for this course? 2. What are your course expectations? 3. Is there anything missing from the course syllabus that you would like to cover this semester? |
| --- |
| **Tool 2:** A Qualitative Post-Assessment Survey   1. Did this course meet your expectations? 2. What did you like about the course? 3. What did you dislike about the course? 4. Would you recommend this course to others in your program? Why or why not? 5. Would you take this course if it was 2 credit hours (with an extra hour of lab each session?) In your opinion, what would be the ideal credit hour(s) for this course? 6. What time of day would you prefer this course? Early morning: 8-9 am; Morning: 9-11 am; Noon: 11-1 pm; Afternoon: 1-3 pm; Late Afternoon: 3-5 pm; Evening: After 5 pm 7. What was the most informative session? 8. What was the least informative session? 9. Was the draft protocol an appropriate final project? Why or why not? |
| **Tool 3:** A Quantitative Student Confidence Post-Assessment Survey  Please circle the appropriate response from the following options: Strongly Agree, Agree, Neutral, Disagree, Strongly Disagree  Self-reported improvements in a specific skill  1. My confidence in my ability to consider appropriate search terms for my research has improved since taking this course.  2. My confidence in my ability to develop a preliminary search strategy has improved since taking this course.  3. My confidence to choose and use a citation management tool has improved since taking this course.  4. My confidence in my ability to assess the risk of bias and reproducibility in scholarly research has improved since taking this course.  5. My confidence in my ability to select appropriate inclusion and exclusion criteria has improved since taking this course.  6. My confidence in my ability to consider if a meta-analysis in an appropriate addition to my systematic review has improved since taking this course.  7. My confidence in my ability to understand the importance of a reproducible and systematic search strategy has improved since taking this course.  8. My confidence in my ability to implement data management strategies has improved since taking this course.  9. I feel more confident in my ability to produce a systematic review now, at the end of the course, than I did when I started the course.  Self-reported confidence in a set of skills   1. I feel confident in my ability to describe the steps in the systematic review process. 2. I feel confident in my ability to select an appropriate database for my research. 3. I feel confident in my ability to consider appropriate search terms for my research. 4. I feel confident in my ability to develop a preliminary systematic review search strategy. 5. I feel confident in my ability to choose and use a citation management tool. 6. I feel confident in my ability to assess the risk of bias and reproducibility in scholarly research. 7. I feel confident in my ability to select appropriate inclusion and exclusion criteria. 8. I feel confident in my ability to consider if a meta-analysis is an appropriate addition to my systematic review. 9. I feel confident in my ability to implement data management strategies. 10. I feel confident in my ability to understand the importance of a reproducible and systematic search strategy. |
