# Supplementary material for: Graduate student confidence following a for-credit systematic review course pilot
Source: J Med Libr Assoc. 2021 Apr 1;109(2):323–9. doi: 10.5195/jmla.2021.1073 (PMC8270359; doi:10.5195/jmla.2021.1073)
Supplement: Supplementary file 2 — Appendix B: Survey Instruments [file jmla-109-2-323-s02.docx]

# ILS 595 – Introduction to Systematic Review for the Health Sciences

## Course Description

This course will introduce systematic review methodology of published health sciences literature. Students will learn to form research questions, develop inclusion and exclusion criteria, search for evidence, manage data, and assess the risk of bias.

## Learning Goals

After completing the course, you will be able to:

- Describe the steps in the systematic review process
- Understand the importance of a reproducible and systematic search strategy
- Identify bias in health sciences literature
- Implement data management strategies

## Required Texts

There is no required text. Readings will come from current literature related to the systematic review process.

## Course Requirements

| **Assignments** | **Points** |
| --- | --- |
| Class Preparation and Participation | 10 |
| Homework I: Find and bring a discipline-relevant systematic review to class. Examine the layout--how is it different from a literature review? | 5 |
| Assignment I: Protocol Part 1-- Choose a framework and develop a research question. Why did you choose your selected framework. | 10 |
| Homework II: Submit a list of 3-4 databases you would search for your systematic review. Justify your choices. | 5 |
| Homework III: Based on the week's readings, consider which keywords and search terms you would include in a search strategy. | 5 |
| Assignment II: Protocol Part 2--Develop a preliminary search strategy | 10 |
| Assignment III: Protocol Part 3--Manage your citations with a citation management tool. Write a paragraph describing your research and use the "Cite While You Write" (or similar) add-on to create in-text citations and generate a biography. | 10 |
| Assignment IV: Protocol Part 4--Select 3 articles and assess the risk of bias for each. | 10 |
| Assignment V: Mock Prospero Protocol | 10 |
| Homework IV: Would a meta-analysis be appropriate for your systematic review? Why or why not? Bring at least 1 question to ask this week's guest speaker. | 5 |
| Final Project: Draft Prospero Protocol | 20 |
| **Total** | **100** |

### *Missed or Late Work*

Assignments will be due at the beginning of the class period on the date that the assignment is due *unless otherwise specified in class or in the syllabus. * Late assignments will be assessed a 10% reduction from full point value, for each day they are late.

## Communication

Communication from me will be through group or individual email to your Purdue Career Account. Email communication will be replied to within 24-48 hours. For best results, please copy both instructors on any emails.

Office hours are by appointment.

## Attendance

Attendance is required. It is recognized that occasionally it may be necessary for a student to be absent from a scheduled course activity for personal reasons beyond their control. You are responsible for class-related work resulting from an unavoidable absence; this work may be made up at our discretion. We may excuse you from a course requirement or responsibility. When conflicts or absences can be anticipated, such as for many University sponsored activities and religious observances, you should inform us of the situation as far in advance as possible. We will strive to accommodate you. For emergencies and other unanticipated events, contact the course instructors.

While in class, students are expected to actively participate during class discussions and activities. Students are encouraged to use their personal laptops or the lab computers as appropriate during class. Please respect the instructors, guest speakers, and fellow students by avoiding cell phone use, unless it is part of the class activity, and putting your phone on vibrate or silence during class. If you are expecting an important call or message during class, please inform the instructor/s before class and wait until you are outside of the room before answering.

All students are expected to have completed any readings and/or pre class assignments before class begins. Students will be required to actively participate during class as part of their participation grade. Participation can include, but is not limited to, sharing with the whole class, sharing in small groups, or completing in class activities.

### Grading Scale

Indicate the numerical ranges for each letter grade. You might also want to include information on whether late assignments will be accepted and any grading penalties for late assignments.

A+ 97 - 100% of points

A 94 - 96% of points

A- 90 - 93% of points

B+ 87 - 89% of points

B 84 - 86% of points

B- 80 - 83% of points

C+ 77 - 79% of points

C 74 - 76% of points

C- 70 - 73% of points

D+ 67 - 69% of points

D 64 - 66% of points

D- 60 - 63% of points

### Incompletes

A grade of Incomplete (I) will be given only in unusual circumstances. To receive an “I” grade, a written request must be submitted prior to February 11, 2019 and approved by the instructor. Requests are accepted for consideration but in no way ensure that an incomplete grade will be granted. The request must describe the circumstances, along with a proposed timeline for completing the coursework. You will be required to fill out and sign an “Incomplete Contract” form that will be turned in with the course grades. Any requests made after the course is completed will not be considered for an incomplete grade.

### Academic Dishonesty

Purdue prohibits "dishonesty in connection with any University activity. Cheating, plagiarism, or knowingly furnishing false information to the University are examples of dishonesty." [Part 5, Section III-B-2-a, [Student Regulations]](http://www.purdue.edu/univregs/) Furthermore, the University Senate has stipulated that "the commitment of acts of cheating, lying, and deceit in any of their diverse forms (such as the use of substitutes for taking examinations, the use of illegal cribs, plagiarism, and copying during examinations) is dishonest and must not be tolerated. Moreover, knowingly to aid and abet, directly or indirectly, other parties in committing dishonest acts is in itself dishonest." [University Senate Document 72-18, December 15, 1972].

Please review the following resource page on plagiarism:

[http://www.education.purdue.edu/discovery/research_integrity.html.](http://www.education.purdue.edu/discovery/research_integrity.html)

For more information on academic integrity please review the below page with Purdue’s student guide for academic integrity:

<https://www.purdue.edu/odos/academic-integrity>

The Purdue Honor Pledge:

“As a boilermaker pursuing academic excellence, I pledge to be honest and true in all that I do. Accountable together - we are Purdue"

## Grief Absence Policy for Students

Purdue University recognizes that a time of bereavement is very difficult for a student. The University therefore provides the following rights to students facing the loss of a family member through the Grief Absence Policy for Students (GAPS). GAPS Policy: Students will be excused for funeral leave and given the opportunity to earn equivalent credit and to demonstrate evidence of meeting the learning outcomes for misses assignments or assessments in the event of the death of a member of the student’s famil*y.*

## Violent Behavior Policy

Purdue University is committed to providing a safe and secure campus environment for members of the university community. Purdue strives to create an educational environment for students and a work environment for employees that promote educational and career goals. Violent Behavior impedes such goals. Therefore, Violent Behavior is prohibited in or on any University Facility or while participating in any university activity.

## Emergencies

In the event of a major campus emergency, course requirements, deadlines and grading percentages are subject to changes that may be necessitated by a revised semester calendar or other circumstances. Relevant changes to this course will be communicated to students electronically. You are expected to read your @purdue.edu email on a frequent basis.

### Accessibility and Accommodations

Purdue University strives to make learning experiences as accessible as possible. If you anticipate or experience physical or academic barriers based on disability, you are welcome to let me know so that we can discuss options. You are also encouraged to contact the Disability Resource Center at: drc@purdue.edu or by phone: 765-494-1247.

## Nondiscrimination

Purdue University prohibits discrimination against any member of the University community on the basis of race, religion, color, sex, age, national origin or ancestry, genetic information, marital status, parental status, sexual orientation, gender identity and expression, disability, or status as a veteran. The University will conduct its programs, services and activities consistent with applicable federal, state and local laws, regulations and orders and in conformance with the procedures and limitations as set forth in [Executive Memorandum No. D-1,](http://www.purdue.edu/policies/pages/human_resources/d_1.html) which provides specific contractual rights and remedies. Any student who believes they have been discriminated against may visit [www.purdue.edu/report-hate](http://www.purdue.edu/report-hate) to submit a complaint to the Office of Institutional Equity. Information may be reported anonymously.

## Class Schedule

| **Session** | **Date** | **Topic** | **Readings** |
| --- | --- | --- | --- |
| 1 | 1/7 | Introduction to course: Student and Instructor  Introductions;  Review  Syllabus; Discussion: Course  Expectations with Bethany McGowan and Jason Reed | PLoS Medicine Editors. (2011). Best practice in systematic reviews: the importance of protocols and registration. *PLoS medicine*, *8*(2), e1001009. |
| 2 | 1/9 | What is a systematic review? Why are systematic review protocols important? with Jason Reed | Grant, M. J., & Booth, A. (2009). A typology of reviews: an analysis of 14 review types and associated methodologies. Health  Information & Libraries Journal, 26(2), 91-108. |

| 3 | 1/14 | Developing a  Research  Question/PICO Framework with Bethany  McGowan | Bragge, P. (2010). Asking good clinical research questions and choosing the right study design. *Injury*, *41*, S3-S6. |
| --- | --- | --- | --- |
| 4 | 1/16 | Selecting appropriate databases and  other literature sources with Jason Reed | Vassar, M., Yerokhin, V., Sinnett, P. M., Weiher, M., Muckelrath, H., Carr, B., ... & Cook, G. (2017). Database selection in systematic reviews: an insight through clinical neurology. Health Information & Libraries Journal, 34(2), 156164. |
| 5 | 1/21 | No class -  Martin Luther  King Jr. Day Holiday |  |
| 6 | 1/23 | Building a search strategy with Bethany McGowan | Hausner, E., Waffenschmidt, S., Kaiser, T., & Simon, M. (2012).  Routine development of objectively derived search strategies.  Systematic reviews, 1(1), 19. |
| 7 | 1/28 | Selecting  Inclusion and  Exclusion  Criteria with  Bethany  McGowan | Meline, T. (2006). Selecting studies for systematic review: Inclusion and exclusion criteria. Contemporary issues in communication science and disorders, 33(21-27). |
| 8 | 1/30 | Useful Tools: Tools for  Screening and  Selecting  Articles with  Jason Reed | Review the following tools and consider which would be practical for your research needs.  *Free:*  Rayyan QCRI: <https://rayyan.qcri.org/welcome>RevMan: [https://community.cochrane.org/help/tools-](https://community.cochrane.org/help/tools-and-software/revman-5)  [and-software/revman-5](https://community.cochrane.org/help/tools-and-software/revman-5)  *Subscription-Based:*  EPPI Reviewer: <http://eppi.ioe.ac.uk/cms/>  Covidence[: https://www.covidence.org/home](https://www.covidence.org/home)  Distiller  SR:  [https://www.evidencepartners.com/products/distillersrsystematic-review-software/](https://www.evidencepartners.com/products/distillersr-systematic-review-software/) |
| 9 | 2/4 | Useful Tools: Citation  Management  Tools with  Jason Reed | Review the following citation management tools and consider which you would use.    Endnote Desktop[: http://guides.lib.purdue.edu/endnote](http://guides.lib.purdue.edu/endnote)    Zotero[: http://guides.lib.purdue.edu/zotero](http://guides.lib.purdue.edu/zotero)    Mendeley:  <http://guides.lib.purdue.edu/c.php?g=352616&p=5860117> |
| 10 | 2/6 | Assessing risk of bias in health sciences literature with  Ellen Wells,  Assistant  Professor of Environmental and  Occupational Health | Drucker, A. M., Fleming, P., & Chan, A. W. (2016). Research techniques made simple: assessing risk of bias in systematic reviews. Journal of Investigative Dermatology, 136(11), e109e114.    Whiting, P., Savović, J., Higgins, J. P. T., Caldwell, D. M., Reeves, B. C., Shea, B., ... & Churchill, R. (2018). ROBIS: a new tool to assess risk of bias in systematic reviews was developed.Journal of Clinical Epidemiology, 69, 225-34. |
| 11 | 2/11 | Mitigating  Bias: Grey  Literature with Bethany McGowan | Godin, K., Stapleton, J., Kirkpatrick, S. I., Hanning, R. M., & Leatherdale, S. T. (2015). Applying systematic review search methods to the grey literature: a case study examining guidelines for school-based breakfast programs in Canada. Systematic reviews, 4(1), 138. |
| 12 | 2/13 | Understanding Meta-analysis with  Yukiko  Maeda,  Associate  Professor of  Educational  Psychology &  Research  Methodology | Shorten, A., & Shorten, B. (2013). What is meta-analysis?. Evidence-based nursing, 16(1), 3-4. |
| 13 | 2/18 | Putting it all together: preparing your Prospero protocol with Bethany McGowan  and Jason  Reed | Protocol Q&A |
| 14 | 2/20 | Final presentations | 8 minutes/student: First 5 Students |
| 15 | 2/25 | Final presentations | 8 minutes/student: Second 5 Students |
| 16 | 2/27 | Course recap | Course assessment and debrief. What did you learn? What would you do differently? Suggestions for future courses? |

### Disclaimer

This syllabus is subject to change. Any changes will be communicated in class and/or via email.
